# A prediction model for good neurological outcome in successfully resuscitated out-of-hospital cardiac arrest patients

**DOI:** 10.1186/s13049-018-0558-2

**Published:** 2018-11-09

**Authors:** Ward Eertmans, Thao Mai Phuong Tran, Cornelia Genbrugge, Laurens Peene, Dieter Mesotten, Jo Dens, Frank Jans, Cathy De Deyne

**Affiliations:** 1 Department of Medicine and Life Sciences, Hasselt University, Diepenbeek, Belgium; 2 Department of Anaesthesiology, Intensive Care, Emergency Medicine and Pain Therapy, Ziekenhuis Oost-Limburg, Schiepse Bos 6, 3600 Genk, Belgium; 3 Interuniversity Institute for Biostatistics and Statistical Bio-informatics, Hasselt University, Agoralaan Gebouw D, 3590 Diepenbeek, Belgium; 4 Department of Cardiology, Ziekenhuis Oost-Limburg, Schiepse Bos 6, 3600 Genk, Belgium

**Keywords:** Out-of-hospital cardiac arrest, Good neurological outcome, Prediction model

## Abstract

**Background:**

In the initial hours after out-of-hospital cardiac arrest (OHCA), it remains difficult to estimate whether the degree of post-ischemic brain damage will be compatible with long-term good neurological outcome. We aimed to construct prognostic models able to predict good neurological outcome of OHCA patients within 48 h after CCU admission using variables that are bedside available.

**Methods:**

Based on prospectively gathered data, a retrospective data analysis was performed on 107 successfully resuscitated OHCA patients with a presumed cardiac cause of arrest. Targeted temperature management at 33 °C was initiated at CCU admission. Prediction models for good neurological outcome (CPC1–2) at 180 days post-CA were constructed at hour 1, 12, 24 and 48 after CCU admission. Following multiple imputation, variables were selected using the elastic-net method. Each imputed dataset was divided into training and validation sets (80% and 20% of patients, respectively). Logistic regression was fitted on training sets and prediction performance was evaluated on validation sets using misclassification rates.

**Results:**

The prediction model at hour 24 predicted good neurological outcome with the lowest misclassification rate (21.5%), using a cut-off probability of 0.55 (sensitivity = 75%; specificity = 82%). This model contained sex, age, diabetes status, initial rhythm, percutaneous coronary intervention, presence of a BIS 0 value, mean BIS value and lactate as predictive variables for good neurological outcome.

**Discussion:**

This study shows that good neurological outcome after OHCA can be reasonably predicted as early as 24 h following ICU admission using parameters that are bedside available. These prediction models could identify patients who would benefit the most from intensive care.

## Background

Despite improvements in advanced life-support and efforts to improve the quality of post-resuscitation care, in-hospital survival after out-of-hospital cardiac arrest (OHCA) remains poor. Within the current post-cardiac arrest (CA) period, outstanding though expensive treatment strategies exist for all comatose patients successfully resuscitated after OHCA [1–4]. Especially within this time period, the uncertain prognosis of OHCA patients fuels the continuous drive of physicians to identify those patients who will benefit the most from aggressive intensive care. Therefore, any argument in favour of good outcome could support the critical decision to use all ICU resources in those patients. Moreover, healthcare workers continuously encounter the optimistic expectations of relatives, and so providing any early information about the likelihood of a good outcome could facilitate communication with patients’ next of kin.

Before the era of targeted temperature management (TTM), a careful interpretation of the clinical neurological examination was considered as the gold standard to determine the prognosis in comatose OHCA survivors [5]. With the implementation of TTM and its concomitant use of sedatives, specific clinical signs have become unreliable for outcome prediction within the initial 24 h [4, 6]. Multiple prognostic markers have been introduced to aid with poor outcome prognostication after OHCA, but do not possess enough discriminatory power on their own to predict outcome (i.e. electroencephalography (EEG), somatosensory-evoked potentials (SSEPs), biochemical markers and brain imaging). Besides, these are not always continuously or sometimes only locally available, are expensive, laborious and above all, require expertise for reliable interpretation [4, 6–8]. Early outcome prognostication should therefore perhaps focus on good rather than poor outcome prediction, especially since guidelines state that the decision to withdraw life-sustaining therapy should be postponed to at least 72 h after CA. Models for the prediction of neurological outcome have been described previously, but use often variables that are rather ambiguous or unavailable at the bedside [9–14]. A prediction model, capable of estimating the probability on good outcome in the early hours based on parameters that are bedside available, could be of major interest for physicians to identify those patients with a reasonable chance of recovery. Additionally, these prediction models might also provide assistance for patient stratification in future randomized controlled trials or epidemiological studies. Therefore, this retrospective study aimed to develop prognostic models – using a training and (internal) validation set – to predict good neurological outcome as soon as possible in OHCA patients using variables that are bedside available after ICU admission.

## Methods

### Study population

All consecutive adult comatose survivors who were successfully resuscitated from OHCA and admitted to the Coronary Care Unit (CCU) of our tertiary care hospital (Ziekenhuis Oost-Limburg, Genk, Belgium), were prospectively enrolled between March 2011 and May 2015. Exclusion criteria were an obvious non-cardiac cause of arrest, in-hospital cardiac arrest and inadequately performed TTM at 33 °C. A head computed tomography (CT) scan was performed if no obvious cause of arrest was found. In this patient cohort, we previously investigated the prognostic value of Near-Infrared Spectroscopy (NIRS) and BIS monitoring, which are neuromonitoring tools known for their non-invasiveness, ease of use and bedside availability [15, 16]. Based on these prospectively gathered data, this retrospective study aimed to construct multivariate prediction models for good neurological outcome using these non-invasive cerebral parameters in conjunction with other variables that are readily available following CCU admission. The study protocol was approved by the local Committee for Medical Ethics (11/066). Written informed consent was obtained from the patients’ next of kin and was reconfirmed if the patient regained consciousness.

### Post-resuscitation protocol

Our institutional post-resuscitation protocol has been described elsewhere [15, 17]. All patients were intubated, mechanically ventilated and sedated by intravenous administration of remifentanil and propofol or midazolam. Unless an obvious non-cardiac cause of arrest could be identified, urgent coronary angiography was performed by interventional cardiologists, followed by a percutaneous coronary intervention. Immediately after admission to the emergency department, TTM at 33 °C was initiated by administering cold saline intravenously (4 °C – 15-30 ml/kg). Once admitted at the CCU, TTM was further mechanically induced and maintained at 33 °C for 24 h using endovascular (Icy-Catheter, Coolgard® 3000, Alsius, Irvine, CA, USA) or surface (ArcticGel™ pads, Arctic Sun® 5000, Medivance, Louisville, Colorado, USA) cooling systems. Hereafter, patients were rewarmed over the next 12 h (0.3 °C/hour). All systems were equipped with a feedback loop system to control target temperature using an oesophageal temperature probe. Only in case of muscle shivering, cisatracurium was administered. Within the TTM period, sedation was titrated to obtain values between − 3 and − 5 on the Richmond Agitation-Sedation scale. Cannulation of the radial artery ensured a continuous registration of blood pressure. Placement of a pulmonary artery catheter was left at the discretion of the treating physician and provided information about mixed venous blood oxygen saturation. According to the guidelines, mean arterial pressure was strictly maintained above 65 mmHg using norepinephrine [18]. Additionally, an hourly blood gas analysis was performed including the determination of lactate. From February 2012 onwards, neuron-specific enolase (NSE) was determined at hour 24 and 48 following CCU admission. Patients were extubated when their neurological, respiratory and hemodynamic status had been recovered sufficiently.

### Neuromonitoring

Cerebral tissue oxygen saturation (SctO_2_) was continuously measured using FORE-SIGHT™ technology (CAS Medical systems, Branford, CT, USA) for 72 h following CCU admission. Furthermore, Bispectral Index (BIS) monitoring using the BIS VISTA™ (Aspect Medical Systems, Inc. Norwood, USA) was started as soon as possible and continued up to 72 h. Both NIRS and BIS sensors were bilaterally placed on the forehead before the start of TTM and covered to prevent ambient light interference. According to manufacturer’s instructions, the BIS sensor was placed above the eyebrows and NIRS sensors were positioned above the BIS sensor. It needs to be stressed that NIRS sensors should not be placed at a place where they are at risk to lose connection with the skin (e.g. on the hairline). Therefore, in patients with a limited amount of space on the forehead to place both NIRS and BIS sensors (due to a lower hairline), priority was given to NIRS, ignorant which of both parameters contained the highest prognostic power. Obviously, this clarifies the high degree of missingness of BIS data in our entire study cohort. Together with hemodynamic data, SctO_2_ was collected with a 2 s time interval and BIS data was stored every second. Although treating physicians were not blinded to the recorded NIRS and BIS values, therapeutic interventions were performed according to the guidelines and at the discretion of the treating physician. As such, the collected NIRS and BIS data were solely being collected for research purposes and were not being used to guide therapeutic interventions or to assist with the process of neuroprognostication.

### Outcome assessment

At 180-days post-CA, surviving patients were interviewed at follow-up by attending cardiologists. These medical reports were retrospectively assessed by a single assessor (W.E.) who defined patients’ outcome using the Cerebral Performance Category (CPC) scale. No outcome data was missing. According to the scale classification, CPC 1 indicates good cerebral performance; CPC 2 signifies a moderate disability but sufficient cerebral functioning for independent daily-life activity; CPC 3 implies severe disability with dependency on others; CPC 4 indicates coma or vegetative state and CPC 5 stands for death [19]. A CPC1–2 and CPC3–5 was considered as a good and a poor neurological outcome, respectively.

### Statistical analysis

**Fig. 1:**
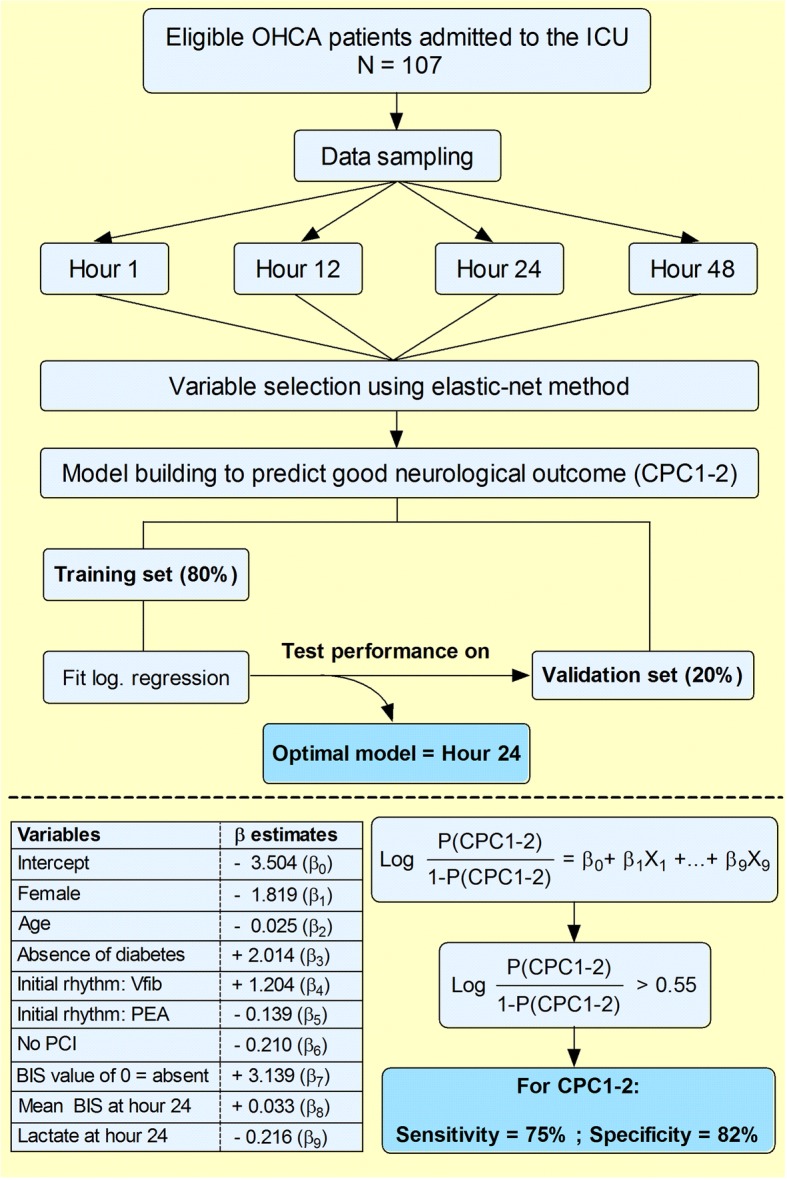
Development of prediction models and calculation used to predict good neurological outcome at hour 24. This flowchart demonstrates the developmental process of the constructed prediction models at selected time points following CCU admission. Twenty-four hours after CCU admission, good neurological outcome was predicted with the lowest misclassification rate (i.e. the optimal model; *top of figure*). The probability for good neurological outcome can be calculated using the correlation coefficients from all variables *(bottom of figure)*. For example, an 84-year old female patient without diabetes, successfully resuscitated from an OHCA with ventricular fibrillation as initial rhythm, was admitted to the emergency department and was transferred to the catherization lab where she received a percutaneous coronary intervention. Twenty-four hours after CCU admission, she did not experienced a BIS value of 0, mean BIS over 24 h was 46 and lactate was 1.2 mmol/l. Based on the formula, the calculated probability of good neurological outcome in this patient would be 0.68 which is higher than the proposed cut-off probability of 0.55. In this specific patient, good neurological outcome can be predicted with a sensitivity of 75% and specificity of 82%

Prediction models for good neurological outcome at 180 days post-CA (CPC1–2) were constructed at hour 1, 12, 24 and 48 after CCU admission (Fig. 1). Variables considered to be included at all time points were: sex, age, diabetes status, witnessed arrest, initial rhythm (with asystole as reference category), percutaneous coronary intervention, initial lactate, initial haemoglobin, initial creatinine, mean arterial pressure, BIS value of 0, mean BIS, mean cerebral oxygen saturation. Along with these variables, the following parameters were considered to be included: lactate, haemoglobin, creatinine and mixed venous oxygen saturation levels at the respective time points. Furthermore, NSE was considered at hour 24 and 48.

To account for missing variables, multiple data imputation was performed. Predictive mean matching imputation was used for continuous variables and logistic regression with bootstrap was performed to impute binary variables. For categorical variables with more than two levels, polytomous logistic regression was used to impute [20]. The number of imputations was equal to the percentage of missingness at each data set for four different time points [21]. The elastic-net method was then used to perform variable selection for all imputed datasets [22]. Variables repeatedly retained in more than 50% of the imputed datasets were chosen for model fitting. To select the optimal values of the elastic-net penalty α and the tuning parameter λ, ten-fold cross-validation was used. The logistic regression model could be specified as:

$$ \mathit{\log}\left[\frac{P\left({Y}_i=1\right)}{1-P\left({Y}_i=1\right)}\right]={\beta}_0+\sum \limits_{j=1}^p{\beta}_1{X}_{ij} $$

Where j (1, p) is the j predictor included in the model and *i* = 1, n is the number of observations in each imputed data set and *P*(*Y*_*i*_ = 1) is the probability of survival for patient *i*.

Once the variables were selected, the performance of the final multivariate logistic regression was assessed for each imputed dataset and results were pooled to make final inference for data at each time point. Each imputed dataset was randomly divided into a training set (80% of patients) and a validation set (20% of patients). Logistic regression was fitted on the training sets and the prediction performance of the resulting model was evaluated on the validation sets by means of misclassification rates (i.e. percentage of cases misclassified; Fig. 1). For this purpose, diverse cut-off points were prespecified. Logistic regression was fitted on all imputed datasets per time point with cut-off points ranging from 0.10 to 0.90 by an increment of 0.05. When the calculated probability from logistic regression was larger than the chosen cut-off point, the patient was categorized as survival (CPC1–2). The corresponding sensitivities and specificities were calculated. Cut-off points that produced both a sensitivity and specificity larger than 70% were chosen. After the cut-off points were determined, the performance of the final (multivariate) logistic regression models constructed at the four time points was assessed by means of the misclassification rate. The optimal cut-off point for each time point was the one with the smallest misclassification rates. Additionally, the area under the receiver operating characteristics curve (AUROC) was calculated for each imputed data set and pooled per time point. We used R 3.2.1 statistical software (R Foundation for Statistical Computing, Vienna, Austria) for multiple imputation, model selection and SAS Software version 13.2 (SAS, Cary, NC, USA) for pooling the results over the different imputed data sets using logistic regression.

## Results

Between March 2011 and May 2015, 147 successfully resuscitated comatose OHCA patients, admitted to the emergency department and transferred to the Coronary Care Unit, were screened for eligibility. Data of 25 patients were excluded due to the following ineligibility reasons: cooling with mattress (*n* = 8), in-hospital cardiac arrest (*n* = 10), drowning/hanging (*n* = 3), no TTM at 33 °C (*n* = 4). Furthermore, 15 out of 122 eligible patients were not retained for final data analysis due to the following reasons: coronary-artery bypass graft surgery at day 2 (n = 1) and not included due to no storage of (continuous) hemodynamic, SctO_2_ and BIS data (*n* = 14). In total, 107 successfully resuscitated comatose OHCA patients with a cardiac cause of arrest were included for data analysis of whom 50 (47%) had a good (CPC1–2) and 57 (53%) a poor neurological outcome (CPC3–5) at 180 days post-CA. Demographic data of all included patients are provided in Table 1. Prediction models for good neurological outcome at 180 days post-CA were constructed at hour 1, 12, 24 and 48 after CCU admission. As two patients died before hour 12, 105 patients were retained for the models at hour 12 and hour 24. Ten patients died between hour 24 and hour 48, resulting in 95 patients who were retained for the model at hour 48.

**Table 1:** Demographics

Parameter	Survivors (CPC1–2)	Non-survivors (CPC3–5)	*P*-value
Patients, n (%)	50 (53)	57 (47)	/
Age, mean (±SD)	61 ± 13	65 ± 13	0.058
Male, n (%)	39 (78)	36 (63)	0.094
Surface cooling, n (%)	25 (50)	36 (63)	0.178
Endovascular cooling, n (%)	25 (50)	21 (37)	0.178
Initial rhythm			
Ventricular fibrillation, n (%)	42 (84)	26 (46)	**< 0.001**
Pulseless electrical activity, n (%)	4 (8)	7 (12)	0.527
Asystole, n (%)	4 (8)	20 (35)	**< 0.001**
Witnessed arrest, n (%)	45 (90)	46 (81)	0.246
Coronary angiography, n (%)	46 (92)	41 (72)	**0.012**
Percutaneous coronary intervention, n (%)	36 (72)	22 (39)	**0.001**
Mean SctO_2_			
At hour 1	64 ± 7	66 ± 6	0.184
At hour 12	65 ± 6	64 ± 5	0.588
At hour 24	68 ± 5	66 ± 6	0.09
At hour 48	71 ± 5	72 ± 6	0.779

Significant values (*p*<0.05) are indicated in bold

In total, 13, 17, 18 and 18 variables were considered in the prediction models at hour 1, 12, 24 and 48, respectively (Table 2). Based on the elastic-net method, 5, 9, 8 and 7 variables were retained in the models at hour 1, 12, 24 and 48, respectively. Variables retained in all prediction models were diabetes, initial rhythm, percutaneous coronary intervention, mean BIS value at the respective time point and the presence of a BIS 0 value within the respective time frames. Lactate and sex were present at hour 12, 24 and 48, while age was only retained at hour 12 and 24 following CCU admission. In addition, creatinine was predictive for good neurological outcome at hour 12 after CCU admission. NSE was determined at hour 24 and 48, but was only retained in the model at hour 48. Mean SctO_2_ values were not present at a single time point (Table 2).

**Table 2:** Prediction models with retained variables at the four time points following ICU admission

Variables	Hour 1 (χ^2^ = 0.95)		Hour 12 (χ^2^ = 0.90)		Hour 24 (χ^2^ = 0.96)		Hour 48 (χ^2^ = 0.99)	
	*Estimate (SE)*	*P-value*	*Estimate (SE)*	*P-value*	*Estimate (SE)*	*P-value*	*Estimate (SE)*	*P-value*
Intercept	−4.462 (1.258)	< 0.001	−1.213 (2.297)	0.598	− 3504 (2.242)	0.118	− 1.124 (1.544)	0.467
Female	–	–	−1.819 (0.843)	0.031	−1.244 (0.763)	0.103	−1.622 (0.939)	0.085
Age	–	–	−0.032 (0.028)	0.245	−0.025 (0.025)	0.332	–	–
Absence of diabetes	1.196 (0.725)	0.099	1.673 (0.982)	0.089	2.014 (0.977)	0.039	1.880 (1.176)	0.110
Initial rhythm								
Ventricular fibrillation	2.213 (0.734)	0.003	0.653 (0.915)	0.475	1.204 (0.872)	0.168	0.717 (0.960)	0.455
Pulseless electrical activity	0.861 (0.972)	0.376	−1.456 (1.234)	0.238	−0.139 (1.228)	0.910	−0.504 (1.387)	0.716
No PCI	−0.776 (0.553)	0.160	−0.630 (0.752)	0.402	−0.210 (0.662)	0.751	−0.315 (0.734)	0.668
Absence of BIS value of 0	1.966 (0.751)	0.009	3.717 (0.942)	< 0.001	3.139 (0.898)	0.001	2.878 (0.942)	0.002
Mean BIS at respective hour	0.017 (0.014)	0.231	0.027 (0.016)	0.085	0.033 (0.019)	0.092	–	–
Lactate at respective hour	–	–	−0.219 (0.187)	0.242	− 0.216 (0.235)	0.358	− 0.136 (0.533)	0.799
Creatinine at respective hour	–	–	−0.331 (0.310)	0.287	–	–	–	–
NSE					–	–	−0.023 (0.016)	0.153

*BIS* Bispectral Index,*NSE* Neuron-specific enolase, *PCI* Percutaneous coronary intervention, *SE* Standard error, *χ*^*2*^ chi-square statistic indicating the goodness-of-fit

These are the final multivariate logistic regression models with retained variables based on the elastic-net method

• Variables considered to be included at all time points: sex, age, diabetes status, witnessed arrest, initial rhythm (with asystole as reference category), PCI, initial lactate, initial haemoglobin, initial creatinine, mean arterial pressure, BIS value of 0, mean BIS, mean cerebral oxygen saturation

• Variables considered to be included at hour 12, 24 and 48: lactate, haemoglobin and creatinine and mixed venous oxygen saturation at respective time points

• Variable considered to be included at hour 24 and 48: NSE at respective time points

Multivariate logistic regression was performed and results were pooled for each time point (Table 2). The pooled χ^2^ of the Hosmer and Lemeshow test for the prediction model at hour 1, 12, 24 and 48 was 0.95, 0.90, 0.96 and 0.99, respectively, indicating a good fit for all models. Then, the performance of all prediction models was assessed by means of the misclassification rate, where the most optimal model is considered as the one generating the lowest misclassification rates. All models predicted good neurological outcome with a sensitivity and specificity above 70% (Table 3). However, the prediction model at hour 24 predicted good neurological outcome with the lowest misclassification rate (21.5%; 95% CI: 19.5–23.5) using a cut-off probability of 0.55 (Mean AUROC = 0.918. Fig. 1).

**Table 3:** Prediction performance of the four prediction models

Cut-off probability	Misclassification rate				Sensitivity				Specificity			
	*H1*	*H12*	*H24*	*H48*	*H1*	*H12*	*H24*	*H48*	*H1*	*H12*	*H24*	*H48*
0.45	26.2 (9.1)	22.9 (8.0)	21.8 (8.2)	–	75.2 (12.5)	78.4 (12.2)	79.8 (12.4)	–	70.8 (14.9)	76.2 (11.8)	77.4 (13.2)	–
0.50	25.3 (9.2)	22.5 (8.2)	21.5 (8.2)	–	72.9 (12.8)	76.5 (12.8)	77.6 (12.9)	–	77.4 (13.7)	78.9 (11.6)	79.9 (12.6)	–
0.55	**24.8 (9.2)**	**22.3 (8.3)**	**21.5 (8.4)**	23.7 (9.6)	70.5 (13.1)	74.1 (13.5)	75.3 (13.6)	78.6 (14.2)	74.3 (14.4)	81.5 (11.3)	82.2 (12.3)	74.6 (15.6)
0.60	–	–	–	23.4 (9.5)	–	–	–	76.8 (14.4)	–	–	–	77.2 (15.0)
0.65	–	–	–	**23.3 (9.4)**	–	–	–	74.6 (14.6)	–	–	–	77.4 (13.2)

Misclassification rate is the percentage of cases misclassified. The optimal cut-off probability yielding the smallest misclassification rate is indicated in bold for each time point. Misclassification rate, sensitivity and specificity are presented in percentage (standard errors)

The probability (P) of survival at hour 24 following CCU admission can be calculated using the following equation:

$$ \mathrm{Log}\ \left[\widehat{\mathrm{P}}\left(\mathrm{Survival}\right)/\left(1-\widehat{\mathrm{P}}\left(\mathrm{Survival}\right)\right)\right]=\kern0.5em {\displaystyle \begin{array}{l}-\kern0.5em \mathbf{3.504}\left(\mathrm{intercept}\right)\\ {}-\kern0.5em \mathbf{1.244}\left(\mathrm{if}\ \mathrm{patient}\ \mathrm{is}\ \mathrm{female}\right)\\ {}-\kern0.5em \mathbf{0.025}\mathrm{x}\ \mathrm{a}\mathrm{ge}\ \mathrm{of}\ \mathrm{patient}\\ {}+\kern0.5em \mathbf{2.014}\left(\mathrm{if}\ \mathrm{diabetes}\ \mathrm{is}\ \mathrm{absent}\right)\\ {}+\kern0.5em \mathbf{1.204}\left(\mathrm{if}\ \mathrm{initial}\ \mathrm{rhythm}\ \mathrm{is}\ \mathrm{ventricular}\ \mathrm{fibrillation}\right)\ast \\ {}-\kern0.5em \mathbf{0.139}\left(\mathrm{if}\ \mathrm{initial}\ \mathrm{rhythm}\ \mathrm{is}\ \mathrm{pulseless}\ \mathrm{electrical}\ \mathrm{activity}\right)\ast \\ {}{}^{\ast } as ystole\  as\  initial\ rhythm\ was\  set\  as\  reference\ category\\ {}-\kern0.5em \mathbf{0.210}\left(\mathrm{if}\ \mathrm{no}\ \mathrm{percutaneous}\ \mathrm{coronary}\ \mathrm{intervention}\ \mathrm{was}\ \mathrm{performed}\right)\\ {}+\kern0.5em \mathbf{3.139}\left(\mathrm{if}\ \mathrm{a}\ \mathrm{BIS}\ \mathrm{value}\ \mathrm{of}\ 0\ \mathrm{was}\ \mathrm{absent}\ \mathrm{within}\ \mathrm{the}\ \mathrm{first}\ 24\ \mathrm{hour}\mathrm{s}\right)\\ {}+\kern0.5em \mathbf{0.033}\mathrm{x}\ \mathrm{mean}\ \mathrm{BIS}\ \mathrm{value}\ \mathrm{a}\mathrm{t}\ \mathrm{hour}\ 24\\ {}-\kern0.5em \mathbf{0.216}\mathrm{x}\ \mathrm{lactate}\ \mathrm{value}\ \mathrm{a}\mathrm{t}\ \mathrm{hour}\ 24\end{array}} $$

Using this cut-off point of 0.55, the prediction model at hour 24 predicted good neurological outcome with a sensitivity of 75.3% (95% CI: 72.1–78.2) and specificity of 82.2% (95% CI: 79.3–85.1) (Fig. 1).

At hour 24, missingness was present in 12 variables, namely initial haemoglobin (0.9%), diabetes (1.9%), witnessed arrest (2.8%), initial Rhythm (3.7%), initial lactate (8.4%), initial creatinine (8.4%), mean MAP at hour 24 (9.5%), mean SvO_2_ at hour 24 (21%), NSE (26.7%), BIS 0 value (27.6%) and mean BIS value at hour 24 (38.1%). Missingness at the other time points is shown in Table 4.

**Table 4:** Percentage of missingness at the four time points following ICU admission

Variables	Hour 1	Hour 12	Hour 24	Hour 48
Mean MAP	11.2%	7.6%	9.5%	23.2%
Mean BIS	34.6%	35.2%	38.1%	46.3%
Absence of BIS 0	33.6%	26.7%	27.6%	25.3%
Mean SvO2	/	21.9%	21.0%	25.3%
NSE	/	/	26.7%	27.4%
Creatinine	8.4%	2.9%	X	9.5%
Lactate	8.4%	X	X	4.2%
Mean SctO2	X	X	X	21.1%

Creatinine and lactate value at hour 1 had the similar percentage of missingness across all time points (both with 8.4%). Missing variables with less than 5% of missingness were initial haemoglobin (1.0%), diabetes (1.9%), witnessed arrest (2.8%) and initial rhythm (3.7%)

*/* variable not included in the respective model

*X* no missingness

## Discussion

Our data show that good neurological outcome at 180 days post-CA can be predicted in successfully resuscitated comatose OHCA patients treated with TTM at 33 °C using prediction models containing variables that are early and bedside available after CCU admission. In order to predict good neurological outcome as early as possible, multilevel prediction models were constructed at hour 1, 12, 24 and 48 after CCU admission which all reached a sensitivity and specificity above 70%. Using a cut-off point of 0.55, the prediction model at hour 24 predicted good neurological outcome with the smallest misclassification rate, corresponding to a sensitivity of 75% and specificity of 82%.

Identifying post-CA patients who would maximally benefit from full supportive therapy without unnecessary suffering remains hard to achieve once admitted to the ICU. Nowadays, specific clinical signs in the initial 24 h have become inaccurate due to the implementation of TTM [4, 6]. Electro-encephalography, SSEPs, biomarkers and brain imaging are prognostic tools recommended by current guidelines to assist with outcome prognostication, but are often not constantly available in daily clinical practice, are time-consuming, expensive and require clinical expertise [4, 23–26]. In an attempt to account for these hurdles and facilitate bedside prognostication, we previously investigated the role of NIRS and BIS monitoring in terms of outcome prediction [15, 16, 27]. This retrospective analysis now aimed to construct multivariate regression models including these cerebral parameters combined with variables, readily available at ICU admission, in order to predict good neurological outcome after OHCA. Unlike scoring systems developed by others, we decided to ignore ambiguous variables such as ‘low-flow’ and ‘no-flow’ times as these are often unknown or incorrectly reported [9–14]. In this study, the constructed prediction models at hour 1, 12, 24 and 48 after admission succeeded to predict good neurological outcome at 180 days post-CA, all with a sensitivity and specificity above 70%. The model which classified OHCA patients with the lowest misclassification errors was the one at hour 24 and contained sex, age, diabetes status, initial rhythm, percutaneous coronary intervention, the absence of a BIS 0 value within the first 24 h, mean BIS value at hour 24 and lactate as predictive variables for good neurological outcome. This model was able to predict good neurological outcome with a sensitivity of 75% and specificity of 82% when 0.55 was used as cut-off point. It has to be stated that the obtained predictive performance of our model should be considered as rather modest. Hence, we certainly do not advise the use of our prediction models to assist with the clinical prognostication process at the moment. On the contrary, external validation in a large patient cohort without missing data will be a prerequisite before clinical implementation will be possible. Additionally, further research attempts should now investigate whether the performance of our constructed prediction models could be improved by adding other prognostic parameters. Therefore, our research findings might be considered as one of the first steps in the development of an easy tool, that is able to identify OHCA patients who might benefit the most from aggressive treatment, and for whom finite healthcare sources should be optimized. For now, our models might be of potential interest as guidance for designing risk stratification models in clinical research with variable resource allocation or could be used to enhance future research initiatives focusing on new therapies. Additionally, the results of this study could be helpful for the design of future epidemiological studies as it is often difficult to select which data should be assembled and when these should optimally be collected after CCU admission [28].

As shown by others, initial rhythm, percutaneous coronary intervention and diabetes status prior to CA were variables retained at all selected time points in this study [29–31]. Likewise, both mean BIS values and the absence of a BIS 0 value appear to be predictors for good neurological outcome across all time points, thereby confirming the prognostic validity of BIS monitoring in the post-CA setting once again [16, 27, 32–34]. In line with previous studies, gender, age as well as lactate and creatinine levels were predictive for good neurological outcome, albeit not immediately following ICU admission [35–38]. Finally, NSE was only retained in the model at hour 48 which is in accordance with previous studies [25, 39].

In recent years, the prognostic value of SctO_2_ has been examined thoroughly in the post-CA setting. Several studies demonstrated that high SctO_2_ values during TTM at 33 °C were associated with a higher likelihood of favourable neurological outcome [17, 40]. Storm and co-authors even suggested a SctO_2_ value of 50% as therapeutic target [41]. In the largest post-resuscitation cohort so far, we previously showed that the overall course of SctO_2_ was different between OHCA patients with a good and poor neurological outcome. Nonetheless, the observed SctO_2_ margin seemed to be too narrow to likely represent outcome differentiation. As such, it was concluded that SctO_2_ lacked prognostic power on its own to serve in outcome prognostication [15]. The role of SctO_2_ as prognostic marker included in a multivariate prediction model, on the other hand, has not been investigated until now. Based on our analysis, we are the first to show that SctO_2_ was not retained in any multivariate regression model at a single time point upon CCU admission. Therefore, this study illustrates once more the limited prognostic value of SctO_2_ by itself in the early hours following ICU admission.

This study has several limitations. First, this was a single-centre study with a limited number of patients included. Secondly, multiple imputation was used to account for missingness in certain variables. Nevertheless, imputed values were deemed as persuasive based on the generated density plots of the observed and imputed data *(not shown)*. On the other hand, a possible selection bias could not have been excluded if only the cases were included with all available parameters. Still, it should be recognised that in some variables, including the SvO2, missingness might not have been completely at random. Although the placement of a pulmonary artery catheter was left at the discretion of the treating physician, it might have been the case that these catheters were placed in clinically more unwell patients, meaning that missing values could have been systematically higher than recorded values. A pulmonary artery catheter was placed in 35 out of the 50 patients with a good neurological outcome (70%) and in 47 out of the 57 patients with a poor neurological outcome (82%). Third, BIS monitoring might not be routinely applied in other centres which might complicate the usefulness of our prediction models. Nonetheless, BIS monitoring is cost-effective, non-invasive and can be made available at the bedside rather easily. On the other hand, BIS data were not kept blinded for treating physicians through which we cannot fully exclude the possibility that the prognostic value of BIS was being artificially inflated during the study period. Nonetheless, treating physicians were cardiologists who are not familiar with the use and interpretation of BIS values. Finally, our prediction models were only validated internally. Even though it has been shown that n-fold cross validation generates stable estimates with low bias, external validation on an independent data set will be mandatory before these models can be used in routine clinical practice [42].

## Conclusion

Prognostic models for the prediction of survival in OHCA patients were constructed at hour 1, 12, 24 and 48 following CCU admission. The prediction model which classified OHCA patients with the lowest misclassification errors was the one at hour 24, yielding a sensitivity of 75% and specificity of 82%. In this model, sex, age, diabetes status, initial rhythm, percutaneous coronary intervention, the presence of a BIS 0 value, mean BIS value and lactate were the variables identified as predictive for good neurological outcome. At the moment, external validation in a larger patient cohort will be mandatory before this model can be translated into clinical practice.

## Abbreviations

BIS: Bispectral index
CA: Cardiac arrest
CCU: Coronary Care Unit
CPC: Cerebral Performance Category
EEG: Electro-encephalography
ICU: Intensive Care Unit
NIRS: Near-Infrared Spectroscopy
NSE: Neuron-specific enolase
OHCA: Out-of-hospital cardiac arrest
P: Probability
SctO_2_: Cerebral tissue oxygen saturation
SSEP: Somatosensory evoked potential
TTM: Tssssargeted temperature management
